# Abiotic Environment Predicts Micro- but Not Macroevolutionary Patterns of Flower Color in Monkeyflowers (Phrymaceae)

**DOI:** 10.3389/fpls.2021.636133

**Published:** 2021-03-25

**Authors:** Dena Grossenbacher, Leah Makler, Matthew McCarthy, Naomi Fraga

**Affiliations:** ^1^Department of Biology, California Polytechnic State University, San Luis Obispo, CA, United States; ^2^California Botanic Garden, Claremont, CA, United States

**Keywords:** anthocyanin, balancing selection, *Diplacus*, drought, *Erythranthe*, floral pigment, *Mimulus*, precipitation

## Abstract

Anthocyanin pigments are responsible for many of the vivid pink, purple, red, and blue flower colors across angiosperms and frequently vary within and between closely related species. While anthocyanins are well known to influence pollinator attraction, they are also associated with tolerance to abiotic stressors such as extreme temperatures, reduced precipitation, and ultraviolet radiation. Using a comparative approach, we tested whether abiotic variables predict floral anthocyanin in monkeyflowers (Phrymaceae) across western North America. Within two polymorphic species, we found that abiotic variables predicted flower color across their geographic ranges. In *Erythranthe discolor*, the frequency of pink flowered (anthocyanin producing) individuals was greater in populations with reduced precipitation. In *Diplacus mephiticus*, the frequency of pink flowered individuals was greater at higher elevations that had reduced precipitation and lower temperatures but less ultraviolet radiation. At the macroevolutionary scale, across two parallel radiations of North American monkeyflowers, species with floral anthocyanins (pink, purple, or red corollas) occupied areas with reduced precipitation in *Erythranthe* but not *Diplacus*. However, after accounting for phylogenetic relatedness, we found no evidence for the joint evolution of flower color and environmental affinity in either clade. We conclude that although abiotic stressors may play a role in the evolution of flower color within polymorphic species, we found no evidence that these processes lead to macroevolutionary patterns across monkeyflowers.

## Introduction

Anthocyanin pigments cause the vivid red, blue, pink and purple colors in flowers, and are one of the most common flower color polymorphisms across angiosperms (Richards, 1986; Warren and Mackenzie, 2001). Floral anthocyanins are widely known for their role in pollinator attraction, reproductive isolation and speciation (e.g., Schemske and Bradshaw, 1999; Zufall and Rausher, 2004; Streisfield and Kohn, 2006; Hoballah et al., 2007). However, many recent studies have found that they also confer tolerance to abiotic stressors such as drought, heat, cold, and ultraviolet radiation, with strong abiotic selection leading to trait-environment correlations (e.g., Warren and Mackenzie, 2001; Coberly and Rausher, 2003; Schemske and Bierzychudek, 2007; Dick et al., 2011; also reviewed in Strauss and Whittall, 2006). This raises the question: are abiotic stressors common drivers of flower color evolution and, if so, does this lead to macroevolutionary patterns such as the association between floral anthocyanin and abiotic stressors across species?

Anthocyanins are the last product in the flavonoid biochemical pathway and likely have an ancient origin—they are present in most land plants including mosses, ferns, gymnosperms, and angiosperms (Campanella et al., 2014). In angiosperms anthocyanins occur in both floral and vegetative tissues, and gene expression is sometimes correlated across tissue types (e.g., Albert et al., 2011; Yuan et al., 2014). Both direct and indirect selection on floral anthocyanins is therefore possible. For example, floral anthocyanins may be directly selected on due to protection of flowers or flower buds from abiotic stressors (e.g., Dick et al., 2011; Koski and Galloway, 2018; Peach et al., 2020) or indirectly through selection in vegetative tissue where anthocyanins protect plants from a variety of biotic and abiotic stressors (Warren and Mackenzie, 2001; Coberly and Rausher, 2003).

While a wide variety of environmental stressors are hypothesized to favor pigmented individuals, three in particular have been the focus of most case studies: drought, thermal extremes and UV radiation. Drought stress occurs when low soil moisture impairs normal plant growth, water relations and water use efficiency (Farooq et al., 2012). Anthocyanin accumulation in plant tissues is known to ameliorate these effects, which means that under low-water conditions, pigmented individuals may have higher fitness than unpigmented individuals (Schemske and Bierzychudek, 2001; Warren and Mackenzie, 2001; Tang and Huang, 2010). For example, in a polymorphic desert annual, *Linanthus parryae*, water use efficiency was greater in individuals with anthocyanin pigmentation (blue flowers) than in those without (white flowers) (Schemske and Bierzychudek, 2001). Fitness estimates across 11 years showed that selection on flower color was strongly correlated with spring precipitation rather than pollinators.

Temperature extremes may also exert selection on floral anthocyanins. For example, in the common morning glory, *Ipomoea purpurea*, high temperatures led to a decrease in male fertility that was greater in individuals without pigmentation than in those with pigmentation (Coberly and Rausher, 2003). Low temperatures (e.g., at or below freezing) may also favor anthocyanin pigmentation in flowers. For example, populations of arctic mustard, *Parrya nudicaulis*, have a higher frequency of purple flowered individuals in locations with lower summer temperatures (Dick et al., 2011). Because floral and vegetative anthocyanin production are not correlated in this species, this could be an example of direct selection on floral pigments due to cold thermal stress. Dark floral pigments have been shown to increase the temperature of a flower by more than 5 degrees C in some species, which could impact pollinator visitation and reproduction (reviewed in van der Kooi et al., 2019).

Finally, high ultraviolet radiation, specifically UV-B, can be detrimental to developing vegetative and floral tissue (Strid et al., 1994; Koski and Ashman, 2015). Anthocyanin pigments may protect tissues from UV-B induced DNA damage by acting as a sort of “sunscreen” (Steyn et al., 2002; Mori et al., 2005). This suggests that anthocyanin pigments should be more common in regions with high UV radiation during the growing season. For example, in *Clarkia unguiculata*, floral anthocyanin concentration was greatest in areas with high UV radiation across its geographic range (Peach et al., 2020).

**FIGURE 1:**
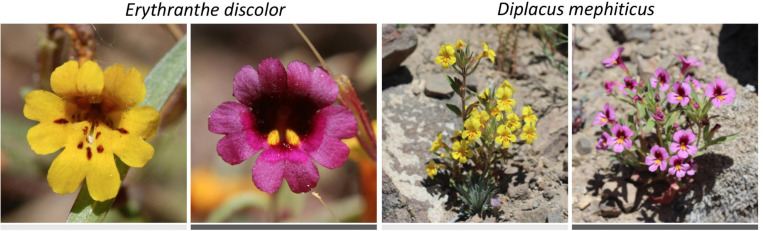
Two focal species that are polymorphic for floral pigments: *Erythranthe discolor* and *Diplacus mephiticus*. Pink-flowered morphs have anthocyanin pigments expressed throughout the corolla tissue, whereas expression is lacking in yellow-flowered morphs.

All of the above examples involve *within*-species comparisons, where both anthocyanin and anthocyanin-less morphs (or continuous variation in anthocyanin concentration in the case of *Clarkia unguiculata*) were present within a single population or species. It is unknown whether these micro-evolutionary processes lead to macroevolutionary patterns across species (but see Dalrymple et al., 2020). Here, we address this question using monkeyflowers (Phrymaceae), a diverse lineage of flowering plants in western North America with incredible variation in floral anthocyanins (Beardsley et al., 2004; Cooley and Willis, 2009; Streisfeld and Rausher, 2009; Yuan et al., 2014) and a wide range of environmental tolerances (Sheth and Angert, 2014). We begin by focusing within two polymorphic species (Figure 1), asking whether the frequency of individuals with pink flowers (caused by anthocyanins) is greater in areas with more drought, thermal stress, or UVB-radiation. We then use a comparative phylogenetic approach to test for correlated evolution of anthocyanin in primary petal tissue and abiotic affinity across 66 species in two major monkeyflower clades, *Erythranthe* Spach. and *Diplacus* Nutt.

## Materials and Methods

### Study System

The family *Phrymaceae* contains over 200 described species of small annuals, perennial herbs, and woody shrubs (Barker et al., 2012; Nesom and Fraga, 2019; Liu et al., 2020). Although it has a worldwide distribution, about 75% of species are restricted to western North America and inhabit a wide variety of environments from deserts in southern California to temperate rainforests in the Pacific Northwest. The two largest genera are *Erythranthe* and *Diplacus* (*N* = 122 and 46 species respectively). Both genera have a center of diversity in the California floristic province and crown ages between 10 and 20 MY (data from Briscoe Runquist et al., 2016). Thus, *Erythranthe* and *Diplacus* represent parallel radiations that occurred during a period of climate change toward hotter drier summers and cooler wetter winters, i.e., during the onset of a Mediterranean climate. Across both clades, present day species occupy a wide range of climates (Grossenbacher et al., 2014; Sheth et al., 2014). While climate affinity is predicted by phylogenetic relatedness across monkeyflowers, there is substantial variation even between closest relatives (Grossenbacher et al., 2014).

Across *Erythranthe* and *Diplacus*, primary flower colors range from pink, purple, yellow, and red to white; many species also have distinctive secondary patterning or nectar guides. In the present study, we focused solely on primary flower color, rather than secondary patterning. In both clades, anthocyanin pigments are responsible for pink and purple corollas, carotenoid pigments for yellow, and a combination of both anthocyanins and carotenoids for red (Vickery and Olson, 1956; Hiesey et al., 1971; Streisfield and Kohn, 2006; Cooley and Willis, 2009; Yuan et al., 2014). While most flower color variation occurs between species, at least eight species are polymorphic for anthocyanin/carotenoid pigmentation with populations containing both pink (anthocyanin) and yellow (carotenoid) flowered morphs (Figure 1 and Supplementary Figure 1).

Flower color has been shown to impact pollinator visitation and pollinator-mediated selection across a number of taxa (e.g., *E. lewisii* and *E. cardinalis*, Schemske and Bradshaw, 1999; *D. aurantiacus*, Streisfield and Kohn, 2006; *E. bicolor* Grossenbacher and Stanton, 2014). Overall, red flowered species are primarily visited by hummingbirds (Schemske and Bradshaw, 1999; Beardsley et al., 2003). Pink, purple and yellow flowered species are primarily visited by bees (Schemske and Bradshaw, 1999; Cooley et al., 2008; Grossenbacher and Stanton, 2014; Kelsey Byers, unpublished data). Moths, butterflies and flies are also important visitors in some taxa (e.g., Streisfield and Kohn, 2006). We know of no studies to date that assess the role of abiotic drivers of selection on flower color in either genus.

### Within Species Comparisons

#### Population Sampling

We focused on two polymorphic annuals that represent the two major lineages in Phrymaceae: *Erythranthe discolor* and *Diplacus mephiticus* (Figure 1). *Erythranthe discolor* is an extremely narrow ranged endemic species in the southern Sierra Nevada, while *D. mephiticus* is widespread across much of the Sierra Nevada and extends into the Great Basin in Nevada. For both species, we defined populations as separate if they were at least 0.5 km apart. In cases where pairs of sites were sampled less than 0.5 km apart (*N* = 2 for *E. discolor* and *N* = 6 for *D. mephiticus*), we randomly removed one member of each pair from all downstream analyses.

In 2017, we conducted field surveys of *E. discolor* populations across a longitudinal gradient that transects the geographic region where both flower color morphs are present. All previously known populations in this region were sampled, as were 3 new populations that were encountered during the 2017 field surveys. At each population, we first identified the spatial extent of all non-flowering and flowering individuals, and then used a 1 by 1 meter grid system to systematically count every flowering individual within the population and recorded whether the primary corolla color was pink (anthocyanin) or yellow (no anthocyanin). The latitude and longitude was recorded from the approximate center of each population. All sampling occurred during the approximate window of peak flowering between June and early July.

To assess flower color in *Diplacus mephiticus*, we utilized herbarium specimens (*N* = 164). We chose to use specimens rather than field surveys mainly due to logistical reasons and to reduce the carbon footprint of our study: *D. mephiticus* populations span hundreds of kilometers and many require multiple day hiking trips. Flower color on each specimen was categorized as either yellow, pink, or polymorphic. Although flower color fades on older specimens, it is usually possible to distinguish pink from yellow. We know this because in many cases corolla color was described on the labels, allowing us to assess our accuracy using a subset of specimens (data not presented). We excluded all specimens where flower color was unclear. Populations that are polymorphic for flower color were often mounted onto separate herbarium sheets (e.g., two sheets with an a or b suffix on the collector number, or in consecutive number series), therefore records were combined and scored as polymorphic if the collector, location and date were identical. Due to the likelihood of undersampling populations that are skewed for a particular color morph, polymorphic populations may have been underscored. The latitude and longitude of each specimen was recorded either directly from the specimen, or by using detailed location information provided on the specimen label to determine the latitude and longitude on a map.

#### Characterizing Environmental Conditions at Each Population

We examined six environmental attributes relating to drought, temperature and UV stressors that vary across our study populations: mean annual temperature, mean annual precipitation, precipitation during peak flower, climatic water deficit, UV-B radiation and elevation. Mean annual temperature and precipitation were extracted from the PRISM dataset using 30 year averages from 1981-2020 (PRISM Climate Group, Oregon State University, http://prism.oregonstate.edu, downloaded June 2020). The PRISM climate interpolation method performs particularly well in mountainous regions where many monkeyflowers occur (Daly et al., 2008). Climatic water deficit (CWD) values were extracted from the TerraClimate gridded data set using 30 year averages from 1981-2020 (Abatzoglou et al., 2018). CWD is a measure of how much energy availability (temperature) exceeds water supply (precipitation), with higher values indicating hotter, drier conditions, and lower values indicating cooler, wetter conditions. UV-B radiation values were extracted from gIUV dataset and represent the sum of monthly mean UVB during the highest quarter (Beckmann et al., 2014). Finally, since temperature, moisture and solar radiation likely vary along elevation gradients, elevation values were extracted from the USGS elevation point query service^1^.

#### Statistical Analysis

To test whether the frequency of pink flowered (anthocyanin present) morphs are predicted by environmental traits across 14 *Erythranthe discolor* populations, we used beta regression models (betareg function in R package betareg, Cribari-Neto and Zeileis, 2009). Beta regression provides a flexible model for continuous response variables defined on the interval (0,1) that display both heteroscedasticity and skewness, e.g., proportional data with many values close to zero. The response variable (frequency of anthocyanin present morphs, y) was transformed prior to analysis, using a standard transformation *y*(*n* – 1) + 0.5/*n* where *n* is the sample size (Smithson and Verkuilen, 2006) because in some cases frequency assumed values of 0 and 1. The six continuous environmental variables were treated as predictors in separate models. Because the response to environmental stressors may be non-linear, we used likelihood ratio tests to compare models with versus without a second order quadratic term and present whichever model was a better fit to our data. Models were fit using maximum likelihood with a bias correction and partial Wald tests were used to determine whether predictors were significant. We accounted for multiple comparisons (*N* = 6), using Benjamini and Hochberg (1995) correction.

To test whether *D. mephiticus* populations that differ in flower color (pink, yellow, or polymorphic) occupy distinct abiotic environments, we used analysis of variance (ANOVA, aov function in base R) or, in cases where model assumptions were not met, the non-parametric Kruskal-Wallis test (kruskal.test function in base R). We again accounted for multiple comparisons (*N* = 6), using Benjamini and Hochberg (1995) correction. In cases where the overall model was significant, we then used a *post hoc* Tukey’s test or the non-parametric Dunn Test to determine which of the three population types occupied significantly distinct abiotic environments (Tukey’s HSD function in base R or dunnTest function in R package FSA, Ogle et al., 2020).

### Between-Species Comparisons

#### Phylogenetic Relationships

We utilized a previously published family level phylogeny (Grossenbacher et al., 2014) which simultaneously estimated the phylogenetic relationships and relative divergence times among *Phrymaceae* species in a Bayesian framework using the nuclear ribosomal ITS and ETS regions and chloroplast *trn*L-F region of Beardsley et al. (2004). All taxonomy was updated to current standards (Nesom and Fraga, 2019) and a sample of 900 trees from the posterior distribution was used for all downstream analyses. Trees were pruned to include only those species for which we obtained flower color and environmental data (see below), resulting in a final dataset of 27 *Erythranthe* species and 29 *Diplacus* species. We note that the topologies of these trees agreed with a recent reconstruction of the *D. aurantiacus* subclade (Stankowski et al., 2019).

#### Characterizing Anthocyanin Presence

To infer anthocyanin presence across species we used flower color descriptions from monographs (Grant, 1924; Thompson, 2005), taxonomic treatments (Nesom and Fraga, 2019), and herbarium specimens. We further corroborated this by visually examining and photographing corollas at multiple populations for all study species (NF and DG made these observations during field work across western North America from 2005-2020). Each species was categorized based on the predominant color of the corolla; nectar guides were excluded. For example, although *E. guttata* often has red spots on the lower corolla lobe, the predominant color is yellow so this was assigned as ‘anthocyanins absent’. This resulted in three potential character states: anthocyanin (red, pink, purple flowers), no anthocyanin (yellow, orange, white flowers), or polymorphic. In cases where the upper and lower corolla lobes differed in predominant color, we assigned the species as ‘anthocyanin’ if either the upper or lower corolla lobes were red, pink or purple (e.g., *E. shevockii*).

#### Characterizing Environmental Affinity

**FIGURE 2:**
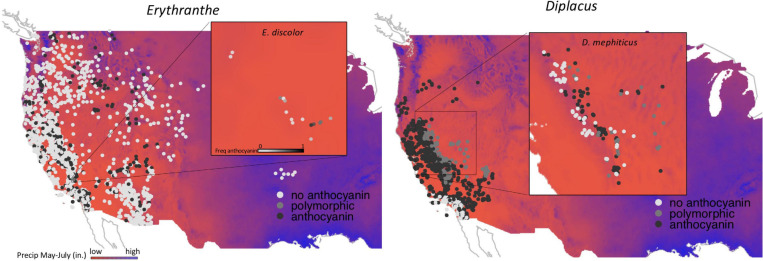
Occurrence data for *Erythranthe* **(left panel)** and *Diplacus* **(right panel)** with filled circle color representing anthocyanin pigmentation (see legend). Background colors represent precipitation during peak flowering with red indicating regions with the lowest precipitation and blue indicating the highest. Insets represent population level data for the two focal species that are polymorphic for flower color.

To identify the average environmental conditions occupied by each species, we utilized a previously curated data set of known occurrences based on herbarium records (Figure 2; Grossenbacher et al., 2014). The average number of occurrences per species was 100 (+ -SE = 30.0, maximum = 1760, minimum = 6). In cases where within-species’ occurrences were within 1 km of one another, occurrences were deleted at random to restrict observations to one record per environmental grid cell in order to reduce sampling bias. We then extracted environmental values for all remaining occurrences and calculated the mean environmental values for each species across all six environmental traits described above.

#### Statistical Analyses

To test whether species that differ in floral anthocyanins occupy distinct abiotic environments, we used ANOVA or, in cases where model assumptions were not met, the non-parametric Kruskal-Wallis test. Species’ floral anthocyanin (anthocyanin, non-anthocyanin, or polymorphic) was treated as a fixed factor and the six environmental traits were treated as response variables in six separate models. We accounted for multiple comparisons (*N* = 6), using Benjamini and Hochberg (1995) correction. In cases where the overall model was significant, we used a *post hoc* Tukey’s test or the non-parametric Dunn Test to determine which of the three anthocyanin categories significantly differed from one another. The same functions and R packages were used here as for the within species analyses described above.

To explicitly test whether there is evidence of correlated evolution of corolla anthocyanins and environmental traits, we used a phylogenetic ANOVA (function aov.phylo, R package geiger, Pennell et al., 2014). Phylogenetic uncertainty was taken into account by performing these tests on a sample of trees from the posterior distribution (*N* = 900). Average *P*-values are reported.

Finally, we tested whether relatedness within *Erythranthe* and *Diplacus* predicts floral anthocyanins, i.e., whether there is phylogenetic signal of flower color. Treating floral anthocyanins as a discrete trait with three character states, we used Maddison and Slatkin (1991) to determine whether the observed number of character state transitions was significantly less than when species names were randomized across the phylogeny (*N* = 999 randomization; R code from Bush et al., 2016). Again, phylogenetic uncertainty was taken into account by performing this test on a sample of trees from the posterior distribution (*N* = 900). Average observed transitions and *P*-values are reported.

All statistical analyses described above were performed in R version 4.0.2 (R Core Team, 2020).

## Results

**FIGURE 3:**
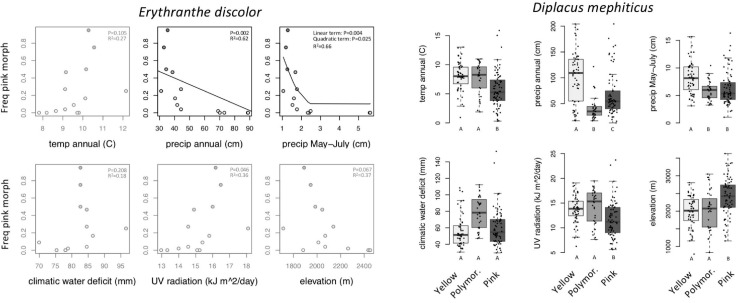
Within-species variation in climate, UV, and elevation affinity by primary flower color category, displayed as boxplots. Filled circles represent observed data for each color category. Lines represent the predicted slope from beta regression and letters indicate *post hoc* comparisons where *P* < 0.05. Opaque plots are those without significant predictors after correcting for multiple tests. See Table 1 for statistical results.

Across 14 populations of *Erythranthe discolor* (Figure 2 inset), the frequency of pink flowered individuals (anthocyanin present) was significantly greater in areas with less annual precipitation and less precipitation during peak flowering May-July (Figure 3 and Table 1). None of the four other environmental variables were significant predictors of flower color after accounting for multiple test comparisons (Table 1).

**TABLE 1:** Results of models analyzing the relationship between abiotic environmental variables and flower color.

	Within species	
Predictor variable:	*Erythranthe discolor* (*N* = 14 populations)	*Diplacus mephiticus* (*N* = 158 populations)
Temp annual	Beta Reg, *Z* = 1.619, *P* = 0.105	Kruskal, *X*^2^ = 25.70, *P* **< 0.001**
Precip annual	Beta Reg, *Z* = −3.239, P = **0.002**	Kruskal, *X*^2^ = 34.39, *P* **< 0.001**
Precip May–July	Beta Reg, linear: *Z* = 2.846, *P* = **0.004**; quadratic: *Z* = −2.58, *P* = 0.025	Kruskal, *X*^2^ = 20.41, *P* **< 0.001**
Climatic water deficit	Beta Reg, *Z* = 1.259, *P* = 0.208	Kruskal, *X*^2^ = 24.53, *P* **< 0.001**
UV radiation	Beta Reg, *Z* = 2.855, *P* = 0.046	Kruskal, *X*^2^ = 20.21, *P* **< 0.001**
Elevation	Beta Reg, *Z* = −1.832, *P* = 0.067	ANOVA, *F* = 11.32, *P* **< 0.001**

	**Between species**	
**Predictor variable:**	***Erythranthe* (*N* = 37 species)**	***Diplacus* (*N* = 29 species)**

Temp annual	ANOVA, *F* = 0.53, *P* = 0.470; phy.anova, *P* = 0.836	ANOVA, *F* = 3.67, *P* = 0.040; phy.anova, *P* = 0.105
Precip annual	ANOVA, *F* = 1.02, *P* = 0.319; phy.anova, *P* = 0.769	ANOVA, *F* = 1.16, *P* = 0.328; phy.anova, *P* = 0.643
Precip May–July	Kruskal, *X*^2^ = 6.32, *P* = **0.004**; phy.anova, *P* = 0.445	Kruskal, *X*^2^ = 5.70, *P* = 0.058; phy.anova, *P* = 0.191
Climatic water deficit	ANOVA, *F* = 5.68, *P* = 0.023; phy.anova, *P* = 0.473	Kruskal, *X*^2^ = 2.52, *P* = 0.284; phy.anova, *P* = 0.723
UV radiation	ANOVA, *F* = 0.47, *P* = 0.499; phy.anova, *P* = 0.845	ANOVA, *F* = 1.23, *P* = 0.309; phy.anova, *P* = 0.460
Elevation	ANOVA, *F* = 2.48, *P* = 0.125; phy.anova, *P* = 0.645	ANOVA, *F* = 4.51, *P* = 0.021; phy.anova, *P* = 0.068

*Note that for phylogenetic ANOVA, we report the mean P-value across 900 Phrymaceae phylogenies sampled from the Bayesian posterior distribution of trees. Bold P-values are significant (P < 0.05) after Benjamini and Hochberg correction for multiple test comparisons.*

Across 158 populations of *Diplacus mephiticus* (Figure 2 inset), flower color was significantly predicted by all six environmental variables (Figure 3 and Table 1). Pink flowered populations occurred in cooler areas with significantly less precipitation than yellow flowered populations, and occurred in areas with less UV radiation and at higher elevations than either yellow or polymorphic populations. Polymorphic populations on the other hand occurred in areas that are both warmer than pink populations, with less precipitation than yellow populations, and had higher climatic water deficit than either pink or yellow populations.

**FIGURE 4:**
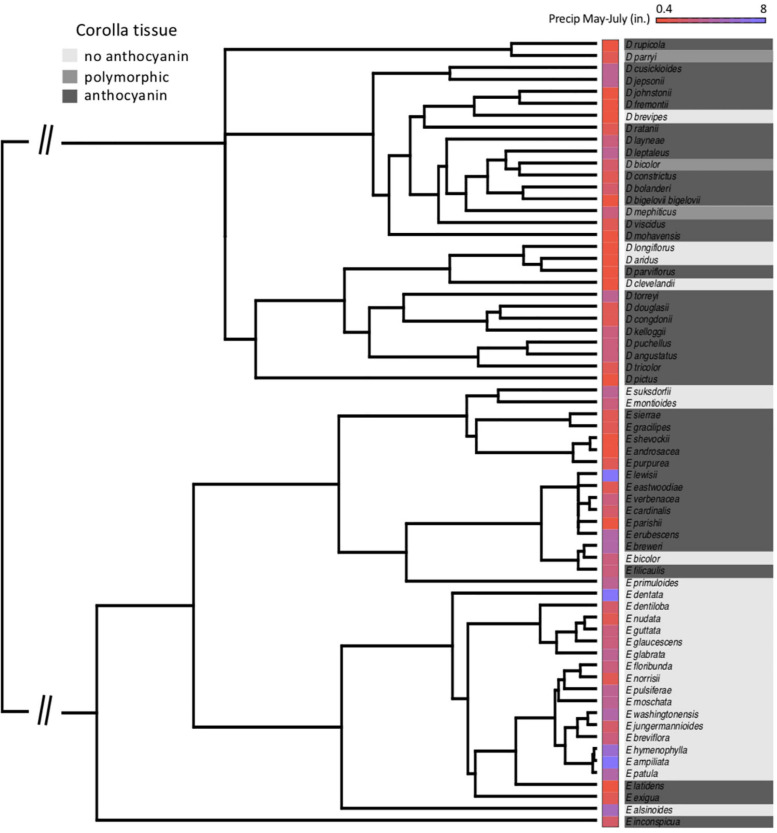
Species’ relationships in clades *Diplacus* and *Erythranthe*, with floral anthocyanin and average precipitation during peak flowering (May–July) indicated. Note that the phylogeny is pruned to only include species in the present study (*N* = 66).

**FIGURE 5:**
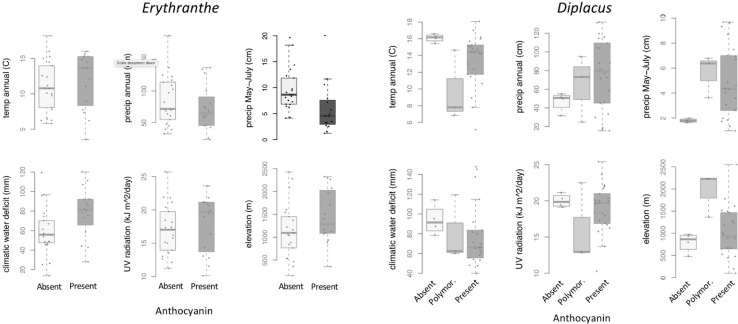
Between-species variation in climate, UV, and elevation affinity by anthocyanin category, displayed as boxplots. Filled circles represent observed data for each anthocyanin category. Opaque plots are those without significant predictors after correcting for multiple tests. See Table 1 for statistical results.

At the species level across the two largest clades of monkeyflowers in western North America (Figures 2, 4), we found that pink flowered *Erythranthe* species occupied areas with significantly less precipitation during peak flower (May-July) than yellow flowered *Erythranthe*, while none of the other traits were significant for either *Erythranthe* or *Diplacus* (Table 1 and Figure 5). We found no evidence of correlated evolution of flower color and any climate traits (phylogenetic ANOVA *P* > 0.05, Table 1).

Finally, we found that floral anthocyanins displayed significant phylogenetic signal in *Erythranthe* but not *Diplacus*. In both clades, there were on average 6 observed transitions in flower color across the posterior distribution of trees—in *Erythranthe*, this was significantly less than when species were randomized across the phylogeny (*P* < 0.001), but not in *Diplacus* (*P* = 0.201).

## Discussion

Our results are consistent with abiotic selection on flower color within populations — flower color in two polymorphic monkeyflowers was correlated with abiotic environmental conditions across the landscape. However, we found no evidence that microevolutionary processes lead to clade-wide macroevolutionary patterns—the evolution of flower color and climate affinity were uncorrelated across monkeyflowers in western North America.

Within two polymorphic monkeyflowers, the frequencies of pink flowers were greater in areas with less precipitation. This is consistent with other polymorphic species where floral anthocyanins were favored in drier environments (e.g., *Linanthus parryae*, Schemske and Bierzychudek, 2001; *Cirsium palustre*, *Digitalis purpurea*, *Holcus lanatus*, *Polygonum persicaria*, and *Vicia sepium*, Warren and Mackenzie, 2001; *Butomus umbellatus*, Tang and Huang, 2010). Together this suggests that across diverse lineages, floral anthocyanins can either directly or indirectly lead to drought tolerance. In heterogeneous landscapes the benefit of anthocyanins may be especially great, and polymorphisms are predicted to be maintained by balancing selection (Warren and Mackenzie, 2001). This may be especially true in the American west, where interannual variation in rainfall is common. While our study examined only two polymorphic monkeyflower species, there are at least six additional species with population-level variation in floral anthocyanins: *E. rubella*, *E. barbata*, *D. leptaleus, D. bicolor*, and *D. parryi*. Future studies using a mix of comparative, experimental and genomic data are needed to uncover the extent of the relationship between anthocyanins, drought stress and balancing selection.

Temperature extremes are another factor that may favor floral anthocyanins. While some previous studies have found that floral anthocyanins protect against high temperatures (Coberly and Rausher, 2003), we found the opposite in *D. mephiticus*—the frequency of pink flowers was greater in areas with *lower* annual temperatures. There is, however, evidence that anthocyanins may also protect plants in near freezing environments (McKown et al., 1996) favoring anthocyanin morphs in cold environments (e.g., Dick et al., 2011). Consistent with this, pink flowered populations of *D. mephiticus* occur at higher elevations than yellow-flowered populations (above 2000 m on average), where near freezing temperatures are more likely to occur during the growing season of this short lived annual species.

Ultraviolet radiation predicted flower color in *D. mephiticus*, but the relationship was opposite what we expected—pink-flowered populations occurred in areas with less UV radiation than yellow-flowered populations. This was surprising given the fact that these populations also occupy higher elevations where UV radiation is generally thought to be more intense but is perhaps explained by more frequent cloud cover. Other studies have also found contradictory patterns regarding UV radiation. For example, in the Neotropical genus *Ruellia*, flavone concentration (which was correlated with floral anthocyanins) was greatest in populations at high latitudes that have correspondingly less UV radiation than populations near the equator (Tripp et al., 2018). The authors concluded that this may be due to flavonoids being selected on by other bioclimatic factors associated with latitudinal gradients, perhaps more so than UV radiation. A similar phenomenon could be occurring with *D. mephiticus.* Environmental factors that are spatially correlated make it difficult to determine the specific factors that drive the evolution of floral anthocyanins. Experimental studies under controlled environmental conditions are needed to tease environmental factors apart and determine their relative importance.

In contrast with the population level patterns described above, we found no support for abiotic drivers of floral anthocyanins at the macroevolutionary scale. We can think of three potential explanations for this finding. First, despite the role of floral anthocyanins in stress tolerance, it could be that pollinator-mediated selection is the stronger agent at the macroevolutionary scale where it can lead to reproductive isolation, speciation and lineage divergence. For example, in the *Diplacus auranticus* species complex, red flowered forms containing anthocyanins and carotenoids are preferred by hummingbirds, while yellow flowered forms containing only carotenoids are preferred by hawkmoths (Streisfield and Kohn, 2006), which could contribute to reproductive isolation and speciation in this complex. Future macroevolutionary studies that jointly examine pollinators and abiotic tolerances are needed to determine their relative contributions to flower color evolution. Second, perhaps the role of anthocyanins in stress tolerance most commonly operates under balancing selection in highly heterogeneous environments, rather than as directional selection. Balancing selection is less likely to lead to changes between lineages. Finally, because flower color is a conserved trait in monkeyflowers, it could be that there is simply low power to detect correlated evolution between flower color and abiotic traits in this system. It is therefore important to explore this question in other clades, perhaps with more frequent transitions in flower color, to determine the role of abiotic selection in shaping floral anthocyanin patterns across angiosperms.

## Data Availability Statement

The raw data supporting the conclusions of this article will be made available by the authors, without undue reservation.

## Author Contributions

All authors contributed to developing the study. NF and MM conducted all within-species data collection and analysis. LM and DG conducted all family-level data collection and analysis. NF, LM, and DG co-wrote the manuscript.

## Conflict of Interest

The authors declare that the research was conducted in the absence of any commercial or financial relationships that could be construed as a potential conflict of interest.

## Footnotes

1 https://ned.usgs.gov/epqs/
