# Supplementary material for: Abiotic Environment Predicts Micro- but Not Macroevolutionary Patterns of Flower Color in Monkeyflowers (Phrymaceae)
Source: Front Plant Sci. 2021 Mar 25;12:636133. doi: 10.3389/fpls.2021.636133 (PMC8030662; doi:10.3389/fpls.2021.636133)
Supplement: Supplementary file 1 [file Image_1.pdf]

Supplementary Material

Abiotic environment predicts micro- but not macroevolutionary patterns of flower color in monkeyflowers (Phrymaceae)

Dena Grossenbacher, Leah Makler, Matthew McCarthy, Naomi Fraga

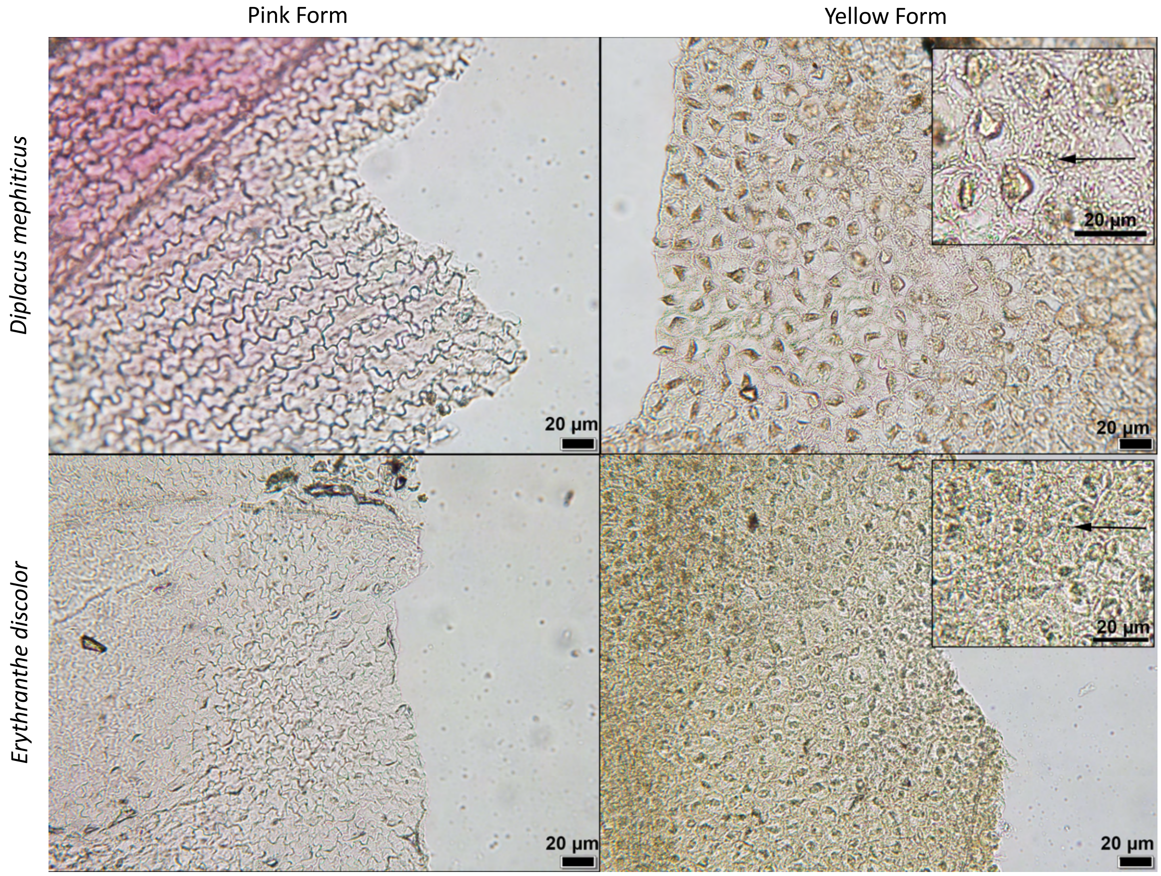

Supplementary Figure 1. Dried petal lobe tissue of pink and yellow morphs of *Erythranthe discolor* (lower panels) and *Diplacus mephiticus* (upper panels). For both species, yellow morphs (right panels) contain chromoplast organelles (indicated with arrows in the inset photos) consistent with presence of carotenoid pigments. In contrast, pink petal morphs (left panels) lack visible chromoplasts. The diffuse pink color in the pink morph of *D. mephiticus* (upper left panel) is consistent with anthocyanin pigmentation in the cell vacuoles. The bottom left image of the pink morph of *E. discolor* was somewhat overexposed which may explain why no color is visible. Voucher specimens associated with images are as follows: *E. discolor* pink form, Fraga #2005 RSA; *E. discolor* yellow form, Fraga #2003 RSA; *D. mephiticus* pink form, Fraga #3758 RSA; *D. mephiticus* yellow form Peirson #11635 RSA
